# Factors Contributing to Rapidly Increasing Rates of Caesarean Section in Andhra Pradesh, India: A Case-Control Study

**DOI:** 10.7759/cureus.37026

**Published:** 2023-04-02

**Authors:** Venkatashiva Reddy B, Sai Subhakar Desu, Rajeev Aravindakshan, Yamini Marimuthu

**Affiliations:** 1 Community and Family Medicine, All India Institute of Medical Sciences, Mangalagiri, Mangalagiri, IND

**Keywords:** trend, audit, emergency, vaginal, caesarean section

## Abstract

Introduction

In some obstetric situations, a caesarean section (CS) can be a crucial, life-saving treatment for both the mother and the infant. Nonetheless, unnecessary CS can raise the risk of morbidity for both. The present study was conducted to study the factors associated with CS delivery and to study the patterns of utilization of health facilities by pregnant women in the state of Andhra Pradesh in India.

Materials and methods

A community-based case-control study was done in Mangalagiri mandal, Guntur district, Andhra Pradesh, India in 2022. A total of 268 mothers (134 CS and 134 normal vaginal childbirth) who delivered between 2019 to 2022 with at least one biological child less than three years of age were studied. The data was collected using a structured questionnaire. Robson’s 10-Group Classification was used to differentiate the type of deliveries of the participants. A p-value less than 0.05 was considered to be significant.

Results

The mean age of the 268 women studied was 25.49±3.73 years. We found that 47 of the 82 (57.3%) women who went to government healthcare facilities and 87 of the 181 (48.1%) women who went to private healthcare facilities had a CS. Of the total CS studied, approximately 83.5% were emergency CSs. All four mothers who had twins had undergone CS. All women with oblique or transverse fetal lie underwent CS irrespective of parity. On multivariate analysis, participants' education status less than or equal to 10th standard was positively associated with CS and identification of complications in the third trimester by healthcare provider was significantly protective for CS.

Conclusion

CS rate reduction necessitates a multi-faceted strategy that includes a variety of programming initiatives. Audits of CS performed as part of health programs and other creative monitoring techniques can be useful tools for assessing the standard of maternity care, particularly emergency CS.

## Introduction

A caesarean section (CS) can be an important, lifesaving procedure for both the mother and the baby in certain medical conditions. However, unnecessary CS can lead to increased medical risks for both mothers and infants [1]. The World Health Organization (WHO) recommends a CS rate of 15% or less to balance the benefits and risks of CS. In addition to the health consequences of high rates of CS, it also puts an additional financial burden on healthcare systems, particularly in low- and middle-income countries. CS is a major surgical procedure and carries health risks for both mothers and infants [1]. Compared with vaginal birth (VB), CS without medical indication is associated with a greater chance of maternal mortality, infection, haemorrhage, adhesions, bleeding and lacerations, bleeding in a subsequent pregnancy, extended hospital stays and/or recovery time, reactions to medication, risk of additional surgeries; and negative emotional reactions for mothers. In addition, infants delivered by CS are at higher risk of having breathing problems, respiratory distress, a low APGAR score, foetal injury, allergic rhinitis, food allergies, childhood asthma, and the childhood onset of type 1 diabetes compared with those delivered by VB [1].

The CS rates in some countries are significantly above the WHO recommendation, e.g., Türkiye (50% of births), Mexico (45%), Chile (45%), Italy (36%), and the United States (32%). In contrast, other countries, including Iceland (15%), Israel (15%), Sweden (16%), and Norway (17%), have CS rates at or near the recommendation [2]. CS rates have increased for women of all ages, races/ethnic groups, and gestational ages in all states of India [3]. According to National Family Health Survey (NFHS-5), 2019-21 in India, more than one-fifth (21.5%) of the deliveries are done by CS, which is slightly higher than the 15% recommendation of the WHO. Moreover, in Andhra Pradesh, the CS rate is 42.4%. Births in private health facilities delivered by CS are 63% and births in public health facilities delivered by CS are 26.6%. According to NHFS-5, 53.8% of the deliveries conducted in the Guntur district were done by CS. CS deliveries in private health facilities are 72.2% and those in public health facilities are 37% [4].

The incidence rates of CS vary widely worldwide. Many countries are taking measures to reduce and/or prevent the increase of CS rates to meet the WHO's recommendation. However, three in 10 women in the United States now give birth by CS. The National Center for Health Statistics reported that the percentage of caesarean births in the United States increased from 20.7% in 1996 to 32.2% in 2014. WHO proposes the Robson classification system as a global standard for assessing, monitoring, and comparing CS rates within healthcare facilities over time, and between facilities. To assist healthcare facilities in adopting the Robson classification, WHO has developed guidelines for its use, implementation, and interpretation, including standardisation of terms and definitions [5].

CS is only recommended when the life of the mother or foetus is at risk. However, this method has currently become a way of escaping from labour pain. People have a common belief that CS is less painful, safer, and healthier than VB. A study reported more than half of women voluntarily undergo CS [6]. Individuals’ interest in experiencing VB, previous positive experiences, lack of anxiety about the safety of mother and baby, faster recovery after delivery, fear of anaesthesia, and attitudes significantly influence the choice of delivery. Economic issues, cultural beliefs and values, previous experiences of childbirth, significant others, and VB facilitators are associated with the choice of VB [6,7]. It seems that cultural norms and beliefs could affect an individual’s tendency towards a certain mode of delivery [8,9]. The present study was conducted to study the factors associated with CS delivery and the patterns of utilisation of health facilities by pregnant women in Andhra Pradesh.

## Materials and methods

This is a case-control study using a simple random sampling technique conducted in 2022 in the Mangalagiri subdivision in district Guntur, Andhra Pradesh, India, among women who delivered a child between 2019 and 2022 with at least one biological child less than three years of age. The participants who were hospitalized, not willing to give consent, or not available after three consecutive visits were excluded from the study. According to the 2011 census, the Mangalagiri subdivision comprises 12 revenue villages. The villages were selected using simple random sampling with a lottery method and all eligible females in the village were enrolled for the study. 

We determined the sample size using James Schlesselman's formula for unmatched case-control [10]. The odds ratio (OR) associated with the exposure that would have sufficient public health importance was hypothesised at 2, and using a 95% confidence interval (CI) and power of 80%, a sample size of 134 in the case group and 134 in the control group was calculated.

A structured pretested questionnaire based on a review of the literature was used. The questionnaire was pretested with 10 women before starting the study. Grammar alterations based on the pretesting were incorporated into the final questionnaire. There were questions on age, educational level, socioeconomic status, marital status, residence, religion, behavioural factors like smoking, other known illnesses, obstetric history, antenatal history, intranatal history, treatment-seeking behaviour, mode of delivery, parity, gestational age, antenatal care attendance, birth weight, referral status, and menstrual history. This was translated into the local language, Telugu, for better understanding by the study participants. The Telugu version of the questionnaire was back-translated into English to check the validity of the form. We defined a "case" as having a CS delivery at the health facilities during the last three years. Controls were women that had a "normal" VB delivery during the last three years. A normal delivery was defined as a spontaneous vaginal delivery.

The list of women delivered in the previous three years in villages of the Mangalagiri subdivision was retrieved from the Center for Rural Health, All India Institute of Medical Sciences (AIIMS), Mangalagiri, Andhra Pradesh, India (also known as Primary Health Centre Nutakkhi). Simple random sampling using the lottery method was done to select the villages. In the selected village, all eligible women were studied. Villages were selected till the sample size was achieved. Anganwadi workers and Accredited Social Health Activists (ASHA) workers served as liaisons to connect to eligible participants. The investigator introduced himself/herself to the study participant before the start of the interview. Participants were given participant information sheets and were informed about the study's objectives, procedures, and rights. When the study participant agreed to participate in the study after going through the information sheet, then a written consent was taken. The study participants were interviewed according to the questionnaire by the study team. The survey was conducted until the final sample size was achieved. The antenatal medical records were reviewed to record the reason for CS and haemoglobin results. All measures to prevent any risk of coronavirus disease (COVID-19) were strictly adhered to in terms of hand washing, mask usage, social distancing, and others.

Robson’s 10-Group Classification was used to differentiate the types of deliveries of the participants. Microsoft Excel 2010 (Microsoft Corporation, Redmond, Washington, United States) was used to enter the data. Analysis was done using IBM SPSS Statistics for Windows, Version 28.0 (Released 2021; IBM Corp., Armonk, New York, United States). Descriptive statistics were conducted for the percentage, mean, and standard deviation (SD); inferential statistics were conducted using chi-square to measure associations between outcomes and explanatory variables, as applicable. unadjusted OR and adjusted odds ratios (aOR) were calculated with 95% CI. A p-value less than 0.05 was considered significant. The multivariate stepwise logistic regression model included covariates that were significant in the bivariate analysis, with a p-value of less than 0.2, and those identified as being associated with CS in the literature.

Ethical approval had been obtained from AIIMS Mangalagiri Ethics Committee (approval number: AIIMS/MG/IEC/2022-23/165 dated June 30, 2022).

## Results

A total of 268 women were studied with 134 normal VB and 134 lower segment (LS) CS deliveries, with a mean age of 25.49±3.73 years and a median age of 25 (interquartile range (IQR) 3). More than half (58.96%) were aged above or equal to 25 years, more than four-fifths (89.18%) were homemakers or not working, almost half (43.28%) were in the middle category in socioeconomic status, and more than half (55.22%) were married at an age of less than 21 years (Table 1).

**Table 1:** Distribution of study participants by sociodemographic characteristics (n=268)

Domain	Category	Mode of Childbirth				Total	
		Vaginal		Caesarean Section			
		n	%	n	%	n	%
Age in years	<25	64	47.76	46	34.33	110	41.04
	≥25	70	52.24	88	65.67	158	58.96
Caste	Other backward caste	82	61.19	82	61.19	164	61.19
	Scheduled caste and scheduled Tribe	52	38.81	52	38.81	104	38.81
Occupation	Working	17	12.69	12	8.96	29	10.82
	homemaker/not working	117	87.31	122	91.04	239	89.18
Education	≤10^th ^Standard	56	41.79	39	29.10	95	35.45
	>10^th ^Standard	78	58.21	95	70.90	173	64.55
Husband's occupation	Manual labour	32	23.88	42	31.34	74	27.61
	Others	102	76.12	92	68.66	194	72.39
Husband's education	≤10^th ^Standard	55	41.04	48	35.82	103	38.43
	>10^th ^Standard	79	58.96	86	64.18	165	61.57
Socioeconomic status	Upper	21	15.67	13	9.70	34	12.69
	Upper middle	36	26.87	51	38.06	87	32.46
	Middle	57	42.54	59	44.03	116	43.28
	Lower middle	16	11.94	11	8.21	27	10.07
	Lower	4	2.99	0	-	4	1.49
Type of the family	Extended	73	54.48	80	59.70	153	57.09
	Nuclear	61	45.52	54	40.30	115	42.91
Age in years at marriage	<21	84	62.69	64	47.76	148	55.22
	≥21	50	37.31	70	52.24	120	44.78
Total		134	50	134	50	268	100.00

**Figure 1:**
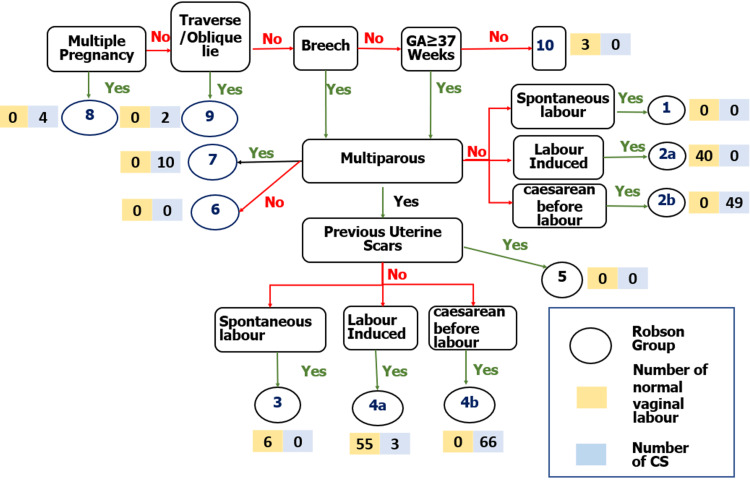
Distribution of study participants by Robson’s classification (n=268) CS: caesarean section; GA: gestational age

Figure 1 shows the distribution of study participants by Robson’s classification. Among the 268 deliveries studied, there were four multiple pregnancies (twins), with all requiring CS. CS has been also performed on all women with an oblique or transverse foetal line, regardless of parity.

Only 2.61% had menarche above the age of 15 years and less than one-tenth (7.84%) had a cycle length other than 28-30 days. Table 2 shows the distribution of participants who had undergone CS delivery with menstrual characteristics.

**Table 2:** Distribution of study participants by menstrual cycle characteristics (n=268)

Domain	Category	Mode of delivery				Total	
		Vaginal		Caesarean			
		n	%	n	%	n	%
Age in years at menarche	≤15	132	98.51	129	96.27	261	97.39
	>15	2	1.49	5	3.73	7	2.61
Cycle length	28-30 Days	125	93.28	122	91.04	247	92.16
	Others	9	6.72	12	8.96	21	7.84
Menstrual flow days	3-5	127	94.78	119	88.81	246	91.79
	Others	7	5.22	15	11.19	22	8.21
Menstrual cycle regularity	Regular	123	91.79	124	92.54	247	92.16
	Irregular	11	8.21	10	7.46	21	7.84
Menstrual cycle-associated problems	Yes	85	63.43	92	68.66	177	66.04
	No	49	36.57	42	31.34	91	33.96
Material used for menstrual flow	Sanitary pads	121	90.30	129	96.27	250	93.28
	Others	13	9.70	5	3.73	18	6.72
Disease present	Yes	3	2.24	3	2.24	6	2.24
	No	131	97.76	131	97.76	262	97.76
Total		134	50	134	50	268	100.00

Less than one-tenth (3.73%) had stillbirths, almost one-fifth (18.66%) had one abortion in the past, less than one-tenth (2.99%) had preterm childbirths, less than one-tenth (1.87%) had home deliveries, more than two-thirds (67.54%) had deliveries at a private institution, more than one-fifth (22.76%) had complications in the third trimester, and almost half (41.79%) had an emergency CS (Table 3). Among the participants, 89.5%, 83.5%, and 61.9% had anaemia in the first, second, and third trimesters of the previous pregnancy, respectively. Forty-seven of the 82 (57.3%) women who went to government healthcare facilities and 87 of the 181 (48.1%) women who went to private healthcare facilities had a CS.

**Table 3:** Distribution of study participants by obstetric characteristics (n=268)

Domain	Category	Mode of delivery				Total	
		Vaginal		Caesarean			
		n	%	n	%	n	%
History of stillbirth	No	128	95.52	130	97.01	258	96.27
	Yes	6	4.48	4	2.99	10	3.73
Number of abortions in past	0	105	78.36	106	79.10	211	78.73
	1	25	18.66	25	18.66	50	18.66
	2	2	1.49	2	1.49	4	1.49
	3	2	1.49	1	0.75	3	1.12
The gestation of childbirth	Full term	129	96.27	131	97.76	260	97.01
	Preterm	5	3.73	3	2.24	8	2.99
Place of delivery	Institutional	129	96.27	134	100.00	263	98.13
	Home	5	3.73	0	0.00	5	1.87
Type of Institutional delivery (n=263)	Government	35	26.12	47	35.07	82	31.17
	Private	94	70.15	87	64.93	181	68.83
Birth weight in kg	2.5-4	118	88.06	115	85.82	233	86.94
	Others	16	11.94	19	14.18	35	13.06
Anaemia in the first trimester	Yes	118	88.06	122	91.04	240	89.55
Complications in the first trimester	Yes	6	11.94	16	11.94	22	8.21
Anaemia in the second trimester	Yes	106	79.10	118	88.06	224	83.58
Complications in the second trimester	Yes	16	95.52	25	18.66	41	15.30
Anaemia in the third trimester	Yes	77	79.10	89	66.42	166	61.94
Complications in the third trimester	Yes	21	20.90	40	29.85	61	22.76
Type of caesarean section	Not applicable	134	100.0	0	0.0	134	50.00
	Emergency	0	0.0	112	83.58	112	41.79
	Elective	0	0.0	22	16.42	22	8.21

**Figure 2:**
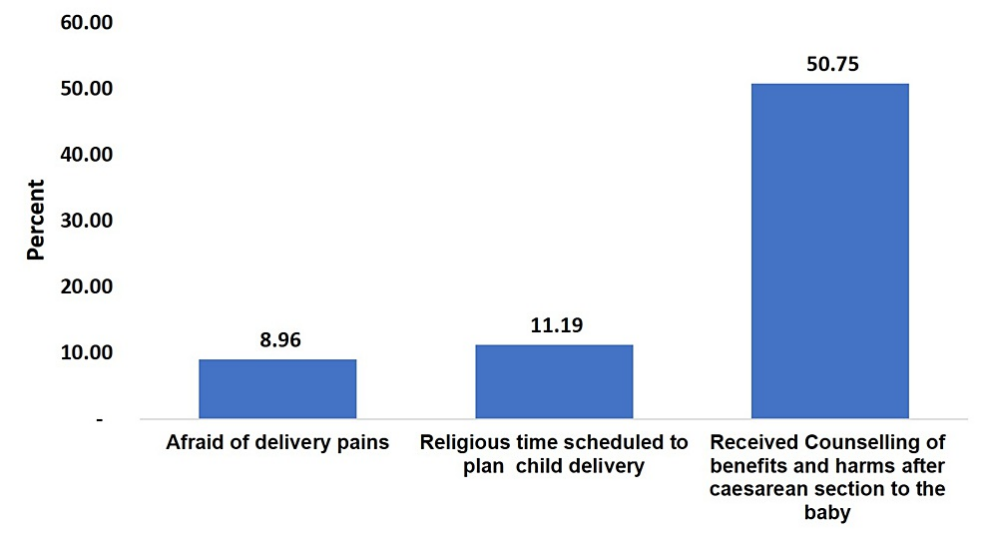
Proportion of selected factors affecting caesarean section (n=134)

Half (50.75%) of participants had received counselling on benefits and harms after caesarean section of the baby, more than one-tenth (11.19%) had a religious belief of an auspicious time for the delivery, and less than one-tenth (8.96%) had informed they were afraid of delivery pains which led to CS (Figure 2). 

Table 4 shows the Robson classification of participants who had a CS. The contribution of Group 2b was 18.28% of the absolute group contribution and more than two-thirds (36.57%) of the relative group contribution. There was a full (100%) group CS rate in groups 2b, 6, 7, and 9.

**Table 4:** Rate of caesarean section by Robson classification group

Robson		Type of delivery		Total (C=A+B)	Group CS Rate % (D=(B/C)*100)	Absolute group contribution to overall CS rate % (H=(B/G)*100)	Relative group contribution to overall CS rate % (I=(B/F)*100)
Group		Vaginal (A)	Caesarean Section (B)				
Group 1	Nulliparous, single cephalic, ≥ 37 weeks, in spontaneous labour	0	0	0	-	-	-
Group 2a	Nulliparous, singleton, cephalic, ≥ 37 weeks' gestation, induced labour.	40	0	40	-	-	-
Group 2b	Nulliparous, singleton, cephalic, ≥ 37 weeks’ gestation, CS before labour.	0	49	49	100.00	18.28	36.57
Group 3	Multiparous (excluding previous cesarean section), singleton, cephalic, ≥ 37 weeks’ gestation, in spontaneous labour.	6	0	6	-	-	-
Group 4a	Multiparous without a previous uterine scar, with singleton, cephalic pregnancy, ≥ 37 weeks’ gestation, induced labour.	85	0	85	-	-	-
Group 4b	Multiparous without a previous uterine scar, with singleton, cephalic pregnancy, ≥ 37 weeks’ gestation, CS before labour.	0	66	66	-	24.63	49.25
Group 5	Previous uterine scar, singleton, cephalic, ≥ 37 weeks’ gestation.	0	0	0		-	-
Group 6	All nulliparous with a single breech.	0	6	6	100.00	2.24	4.48
Group 7	All multiparous with a single breech (including previous CS).	0	10	10	100.00	3.73	7.46
Group 8	All multiple pregnancies (including previous CS).	7	4	11	36.36	1.49	2.99
Group 9	All women with a single pregnancy in transverse or oblique lie (including those with previous CS).	0	2	2	100.00	0.75	1.49
Group 10	All singleton, cephalic ≤37 weeks (including previous CS).	3	0	3	-	-	-
Total		134 (E)	134 (F)	268 (G)	50.00	50.00	100.00

On bivariate analysis with an unadjusted OR of 1.74 (1.07 to 2.86), 1.74 (1.05 to 2.90), 1.83 (1.12 to 2.99), and 2.02 (1.09 to 3.71), a positive association of CS was found with age less than 25 years, education less than or equal to 10th standard, age at marriage less than 21 years, and temporary type of house, respectively (Table 5). On multivariate analysis, participants' education status less than or equal to 10th standard was positively associated with CS with an aOR of 2.22 (1.16 to 4.24) (p=0.016). Identification of complications in the third trimester by healthcare provider was significantly protective for CS with an aOR of 0.31 (0.12 to 0.86) (p value=0.025). 

**Table 5:** Univariate and multivariate analysis for factors affecting caesarean section ^ Reference value *Statistically significant P-value <0.05

Domain	Category	Univariate			Multivariate	
		Unadjusted OR	chi-square	P-value	Adjusted OR	P-value
Age in years	≥25^					
	<25	1.74 (1.07 to 2.86)	4.996	.025^*^	1.36 (0.76 to 2.45)	0.294
Cast	Scheduled Caste/Scheduled tribes ^					
	Other Backward classes	1.0 (0.61 to 1.63)	0	1	1.04 (0.60 to 1.81)	0.874
Occupation	House wife/not working^					
	Working	1.47 (0.67 to 3.27)	0.967	0.326	..	..
Education	>10th standard^					
	≤10th standard	1.74 (1.05 to 2.90)	4.713	.030^*^	2.22 (1.16 to 4.24)	0.016*
Husband Occupation	Others^					
	Manual Labour	0.68 (0.40 to 1.17)	1.867	0.172	0.32 (0.16 to 0.66)	0.002*
Husband education	>10th standard^					
	≤10th standard	1.24 (0.76 to2.04)	0.773	0.379	..	..
Socioeconomic status	Middle or Lower^					
	Upper	0.81 (0.50 to 1.31)	0.738	0.39	..	..
Type of the family	Nuclear^					
	Extended	0.81 (0.49 to 1.31)	0.746	0.388	..	..
Age in years at marriage	≥21^					
	<21	1.83 (1.12 to 2.99)	6.036	.014^*^	1.62 (0.88 to2.97)	0.116
Type of house	Permanent^					
	Temporary	2.02 (1.09 to 3.71)	5.147	.023^*^	1.87 (0.95 to 3.66)	0.068
The complication in First Trimester	No^					
	Yes	0.34 (0.13 to 0.91)	4.95	0.026*	0.27 (0.70 to 1.08)	0.06
The complication in Second Trimester	No^					
	Yes	0.59 (0.3 to 1.16)	2.33	0.12	2.94 (0.78 to 11.09)	0.112
The complication in Third Trimester	No^					
	Yes	0.43 (0.24 to 0.79)	7.662	0.006*	0.31 (0.12 to 0.86)	0.025*
Type of delivery Institute	Private^					
	Government	0.68 (0.41 to 1.16)	1.93	0.16	..	..

## Discussion

We investigated factors associated with CS delivery in the Mangalagiri subdivision of Guntur district, Andhra Pradesh. The age of the mother was discovered to be a poor predictor of CS delivery. Research, on the other hand, shows that the mother's age may be linked to the likelihood that she will have a CS [11]. Older mothers (over the age of 35) and younger mothers (under the age of 20) were more likely to have a CS compared to mothers in their 20s and early 30s. This link is caused by a number of things, including changes in the mother's body and a higher risk of medical problems that could lead to a CS being needed. On the other hand, younger mothers may have small pelvises, or their foetus may be more likely to be malpositioned, both of which can make it more likely that they'll need a CS [11]. This could have been because of the study's participants' relatively younger age distribution, with fewer women over the age of 30 and younger than 18 years.

Studies have found a strong link between a woman's level of education and her likelihood of having a CS during childbirth [12-14]. We found a positive association between mothers having an education status less than or equal to the 10th standard and the risk of CS. Similarly, a study by Eliner et al. has shown that women with higher education levels are less likely to have CS than those with lower education levels [15]. This could be due to several reasons, including the fact that women with higher education levels tend to have more resources and better access to healthcare, which can help them manage their pregnancies better and avoid the need for CS, women with higher education levels may have a better understanding of pregnancy and childbirth, which can help them make informed decisions and communicate effectively with their healthcare providers, and women with higher education levels may be more likely to engage in healthy behaviours such as regular exercise and good nutrition, which can help them have a healthy pregnancy and avoid complications that may require a CS.

In the current study, it was found that women whose husbands were from the labour class had a lower risk of having a CS. On the contrary, the study by Milcent and Zbiri found that women with lower socioeconomic status, as indicated by their husbands' occupations, were more likely to have CS than those with higher socioeconomic status. According to the study, this could be due to factors such as less exposure to prenatal education [16]. Another study found that women whose husbands were manual labourers were more likely to have CS compared to those whose husbands had professional occupations. The study suggested that this association might be due to factors such as limited access to prenatal care and increased stress levels, which could affect the health of the mother and the baby and increase the likelihood of needing a CS [17]. This highlights that there may be a significant relationship between a woman's husband's occupation and the likelihood of having a CS. However, other factors such as maternal health and access to healthcare also play a role in determining the need for a CS during childbirth.

We discovered that detecting complications in the third trimester by healthcare providers was statistically associated with a lower risk of CS. A study mentioned that by identifying women at risk for CS early in pregnancy, healthcare providers can take steps to manage these risk factors and increase the likelihood of successful VB [18]. Additionally, early identification of risk factors can help healthcare providers plan for a safe delivery and ensure that the appropriate resources are available to manage any potential complications. This can help reduce the likelihood of emergency CS and improve outcomes for both the mother and the baby.

We found that 47 of the 82 (57.3%) women who went to government healthcare facilities and 87 of the 181 (48.1%) women who went to private healthcare facilities had a CS. There is evidence to suggest that the rates of CS are nearly similar in government and private health facilities. A study conducted in India found that the overall rate of CS was similar in both government and private health facilities, with a rate of 23.5% in government facilities and 27.6% in private facilities. However, the study also noted that the indications for CS were different in government and private facilities, with a higher proportion of CS performed for non-medical reasons in private facilities [19]. Another study conducted in Egypt found similar rates of CS in government and private facilities, with rates of 35% and 36%, respectively. However, the study noted that there were differences in the characteristics of women who gave birth in government and private facilities, with women in private facilities being more likely to have higher education and higher income [20]. Overall, while the rates of CS may be similar in government and private health facilities, it is important to note that there may be differences in the reasons for performing CS and the characteristics of women who give birth in these facilities.

In the present study, of the total CS, approximately 83.5% were emergency. This high rate of emergency CS despite appropriate antenatal care suggests that an increasing rate of emergency CS may not always be medically indicated. Several studies have reported that a significant proportion of emergency CS is performed in situations where VB may have been a safe and reasonable alternative. A study found that up to 40% of emergency CS performed in the United Kingdom (UK) may not have been medically necessary [21]. Another study, by Palatnik and Grobman, found that up to 50% of emergency CS performed in the United States may have been avoidable with appropriate obstetric care [22]. There are several possible reasons for the high rate of medically unnecessary emergency CS. Doctors are among those who are more likely to perform an emergency CS in order to avoid potential legal liability in the event of a negative outcome. In some cases, doctors may not wait for labour to progress naturally and instead plan for an emergency CS, even when there is no immediate threat to the health of the mother or the baby. In some cases, women may have already requested an elective CS for non-medical reasons, but then experience complications during labour that necessitate the emergency CS [23,24].

To make sure that CS is only done when they are needed, doctors should follow evidence-based guidelines and involve patients in making decisions. This means talking to the patient about the risks and benefits of each delivery method and helping them make an informed choice that takes their situation and preferences into account. CS audits by the government can be a good way to keep track of emergency CS and make sure they are only done when they are medically necessary. Such audits can help identify hospitals or healthcare providers that have higher rates of CS or avoidable CS and can provide data to inform quality improvement initiatives. In the UK, the National Institute for Health and Care Excellence recommends that all hospitals and maternity units undertake regular audits of their CS rates and outcomes, including both elective and emergency procedures. These audits should be used to identify areas for improvement, such as reducing rates of elective CS, increasing rates of VB after a previous CS, and improving clinical decision-making around emergency CS. In addition, the UK government has introduced initiatives to improve the quality of maternity care and reduce the rate of unnecessary interventions, such as the Better Births initiative and the National Health Service (NHS) Maternity Transformation Programme. [25] These initiatives aim to promote evidence-based practices, improve communication and collaboration among healthcare providers, and empower women to make informed decisions about their care. In addition to CS audits, there have been other initiatives in India aimed at reducing the rate of unnecessary CS, such as the 'Birth Checklist' programme, which is being implemented in select states. This programme provides a checklist of evidence-based practises for safe and effective childbirth, including the appropriate use of CS, and is designed to improve the quality of maternal and newborn care [26].

There are a few limitations of the present study. First, the matching of cases and control could not be done due to logistic reasons. Second, the age recorded of participants and age at marriage were self-reported so there can be minor variation in the mean age of participants and the mean age at marriage.

## Conclusions

A total of 268 women were studied in the present study. We found the rates of CS were near equal in the participant accessing healthcare during pregnancy in government or private health facilities. About 83.5% of the CSs were emergency operations. CS rate reduction necessitates a multi-faceted strategy that includes a variety of programming initiatives. Audits of Caesarean sections performed as part of health programs and other creative monitoring techniques can be useful tools for assessing the standard of maternity care, particularly emergency CS. The study found that the lower education level of mothers, and wives of non-labour husbands had a higher risk of CS. This necessitates the need for continuous health education about labour during the antenatal period among expectant mothers.
